# Construction Sheets Made of High-Performance Flame-Retardant Nonwoven Fabrics and Combustion-Resistant Polyurethane Foam: Preparation Process and Property Evaluations

**DOI:** 10.3390/polym15040953

**Published:** 2023-02-15

**Authors:** Bing-Chiuan Shiu, Chen-Hung Huang, Hua-Lin Yang, Yueh-Sheng Chen, Ching-Wen Lou, Jia-Horng Lin

**Affiliations:** 1 College of Material and Chemical Engineering, Minjiang University, Fuzhou 350108, China; 2 Department of Aerospace and Systems Engineering, Feng Chia University, Taichung 407102, Taiwan; 3 Fujian Key Laboratory of Novel Functional Fibers and Materials, Minjiang University, Fuzhou 350108, China; 4 Laboratory of Fiber Application and Manufacturing, Advanced Medical Care and Protection Technology Research Center, Department of Fiber and Composite Materials, Feng Chia University, Taichung 40724, Taiwan; 5 Department of Bioinformatics and Medical Engineering, Asia University, Taichung City 413305, Taiwan; 6 Department of Biomedical Engineering, China Medical University, Taichung 404333, Taiwan; 7 Department of Medical Research, China Medical University Hospital, China Medical University, Taichung 404327, Taiwan; 8 School of Chinese Medicine, China Medical University, Taichung 404333, Taiwan; 9 Innovation Platform of Intelligent and Energy-Saving Textiles, School of Textile Science and Engineering, Tiangong University, Tianjin 300387, China; 10 Advanced Medical Care and Protection Technology Research Center, College of Textile and Clothing, Qingdao University, Qingdao 266071, China

**Keywords:** nonwoven fabrics, polyurethane (PU) foam, electromagnetic interference shielding efficacy (EMI SE), aluminized foil film, Basalt woven fabrics

## Abstract

In this study, nonwoven fabrics, rigid polyurethane foam (RPUF), Basalt woven fabrics, and an aluminum foil film mold are used to produce multi-functional composite sheets with flame-retardant, sound-absorbing, and electromagnetic-shielding functions. The nonwoven layer is composed of Nomex fibers, flame-retardant PET fibers, and low-melting-point (LMPET) fibers via the needle rolling process. The optimal Nomex fiber/flame-retardant PET fiber/LMPET fiber (N/F/L) nonwoven fabrics are then combined with rigid polyurethane (PU) foam, Basalt woven fabric, and an aluminum foil film mold, thereby producing nonwoven/rigid polyurethane foam/Basalt woven fabric composite sheets that are wrapped in the aluminized foil film. The test results indicate that formed with a foaming density of 60 kg/m^3^ and 10 wt% of a flame retardant, the composite sheets exhibit electromagnetic interference shielding efficacy (EMI SE) that exceeds 40 dB and limiting oxygen index (LOI) that is greater than 26. The efficient and highly reproducible experimental design proposed in this study can produce multifunctional composite sheets that feature excellent combustion resistance, sound absorption, and EMI SE and are suitable for use in the transportation, industrial factories, and building wall fields.

## 1. Introduction

With the development of society, urban areas are becoming more highly populated with heavy traffic and ubiquitous high-rises. As the standard of living is increasing, people are emphasizing personal safety to a greater extent [1,2,3] and becoming conscious of personal protection and environmental issues, e.g., noise hazard, fire, and non-atomic ionizing radiation [4]. From the perspective of environmental pollution, large epidemiological studies prove to relate two facts: that the public are exposed to environmental noise and that they are inflicted with cumulative adverse health effects [5,6,7]. Despite this great potential for sandwich construction, there are legitimate concerns associated with the mechanical performance of polymeric foams when subjected to elevated temperatures or fire; due to their organic nature, these foams are combustible and flammable. Moreover, at approximately 300 °C, they undergo thermal decomposition, releasing toxic gases (e.g., isocyanate and amines in case of polyurethane) and smoke [8]. Moreover, pathological disorders can be attributed to long-term exposure to environmental noise caused by a combination of audible/inaudible spectra of artificial sound sources and sound-induced vibrations. Sonic overload arouses the perception–reaction of the auditory system, affecting the whole central nervous system [9,10,11]. Regardless of whether it is considered as noise or from a privacy protection perspective, it is imperative to develop composite sheets that are sound absorbent and small in size to satisfy the requirements of interior design decoration. Sound absorption materials in current use perform inadequately, and different materials also exhibit common disadvantages in practical application, such as an inflammability attribute, rapid spread of flame, hazard of toxic dense smoke, or the presence of airborne micro/nanofibers during the construction process that irritate the skin or jeopardize the lungs [7,12,13,14]. Some materials react with a short service life and low durability in a moist environment [15,16]. For example, polyurethane (PU) foam planks are used primarily as a construction material, whereas they demonstrate poor flame-retardant properties and thermal stability characteristics. To compensate for this, sound-absorbing polymer materials are incorporated in order to mechanically strengthen the PU foam [17].

Zhewen Ma et al. (2022) produced flame-sensitive sandwiched polyurethane flame-retardant composite via stick coating, spray coating, and brush painting. The proposed efficient versatile ternary coating strategy represents a new prospect of the development of flame-retardant polymer foams that are used in construction, traffic, channels, and public areas [18]. Namely, they used polyamide (PA), flame-retardant copolymer, graphene oxide, and carbon nanotubes (CNT) with the sandwich method, stick coating, spray coating, and brush painting techniques, thereby forming PU foam with a flame-retardant composite coating [19]. Li et al. (2021) also applied a coating to strengthen the polyethylene terephthalate (PET) fabrics. Ammonium polyphosphate, polydopamine, silica, and silver nanoparticles are composites coated the PET fabrics, improving the flame-retardant performance [20]. Nevertheless, materials with single composition or single functionality are no longer adequate to satisfy the requirements for construction materials in their current state. As a result, the current trend in recent studies is to apply the composite material structure to multifunctional feature sheets. It is highly expected that the functional composites will be continually developed to better the living environment with regard to comfort, function, and safety [21,22].

Based on the living space, the composite sheets in this study are pertinent to the construction fields, such as industrial plant compartment and basic building walls, roofs, and floors. The staple material is rigid polyurethane foam that accounts for 85% of the composites, and, concurrently, nonwoven fabrics, woven fabrics, and aluminized foil films are combined for the pursuit of flame retardance, heat insulation, and reduction of noise and moisture, and shielding electromagnetic waves functions. Multi-functional rigid PU composite planks are being developed with different composition proportions for the corresponding required functions. One surface layer of the composite planks is composed of a nonwoven fabric, with the opposite surface layer being an aluminized foil film. In addition, a PU foam layer and Basalt woven fabrics are used as the core layer. The porous fiber structure of nonwoven fabrics attenuates the progress of acoustic noise and vibration energy. On one hand, the incident sound waves enter the cells of the PU foam and are then transformed into thermal energy and lose viscous flow due to the reflection and boundary layer friction, thereby insulating the air [23,24,25,26]. On the other hand, vibration energy enters the cells of the PU foam to cause resonance of cavity, which, in turn, attenuates the energy, thus attaining the sound absorption and shock absorption functions [27,28,29]. Taking advantage of the acoustic absorption efficacy of nonwoven fabrics and rigid PU foam, this study changes the blending ratio of nonwoven layer, thereby examining the effect of the blending ratio as related to the frequency of sound waves as well as sound absorption efficacy. In addition, aluminized foil film is incorporated during the foaming process, which effectively reduces the interference of excessive electromagnetic waves in the proximity. This easy measure enriches the functions for composite planks. In particular, the nonwoven layer is composed of Nomex fibers, low-melting-point PET fibers (LMPET), and flame-retardant PET fibers via the needle rolling technique, during which the fiber blending ratio is changed in order to form a nonwoven layer with good functions. Moreover, rigid PU foam is incorporated with a flame retardant to strengthen the rigid PU foam composites in terms of combustion resistance. The combination of basalt woven fabric not only greatly increases the mechanical strength but also reduces the damaged area of PU foam during combustion. Similarly, the combination of basalt woven fabric not only greatly increases the mechanical strength but also reduces the damaged area of PU foam during combustion. This research is based on self-made flame-retardant PU foam and flame-retardant nonwoven fabric with the best ratio, adding aluminized foil film and basalt woven fabric to greatly increase its function.

## 2. Experimental Section

### 2.1. Materials

Recycled Nomex^®^ fibers (Fumao Industrial Co., Ltd., Taichung, Taiwan), flame-retardant PET fiber (FR-PET), and low-melting-point polyester fiber (LMPET) are used. Basalt woven fabrics (Far East New Century Co., Ltd., Taoyuan, Taiwan) have a plain weave and a fineness of 330 tex. Also used are flame retardant (FR-047, Guang Long Hsing Co., Ltd., Taichung, Taiwan), aluminized foil film (Taiwan Vacuum Coating Co., Ltd., Taichung, Taiwan), and two-part type rigid foam (Zhongxing Chemical Materials Co., Ltd., Tainan, Taiwan): the main component of Part A is polyol (Polyol), with the specific gravity of 1.12–1.15 g/cm^2^ and a viscosity of 900 ± 100 CPS/25 C, and the main component of agent B is diphenylmethane diisocyanate, with the specific gravity of 1.23–1.24 g/cm^2^ and a viscosity of 200 ± 50 CPS/25 C.

### 2.2. Preparation of Flame-Retardant Nonwoven Fabrics

All the fibers are processed, with opening and impurity removal performed in advance. Nomex fibers (30, 40, and 50 wt%), flame-retardant PET fibers (50, 40, and 30 wt%), and LMPET fibers (20 wt%) are blended at different ratios. With a specified amount of LMPET fibers, the main focus is on the optimal Nomex/PET blending ratio. Moreover, different blends are processed with the needle rolling density of 1.25, 1.5, and 1.75 needles/cm^2^ to form matrices. Finally, a hot-pressing machine is used to bond the nonwoven fabrics at 120, 140, or 180 °C. Nonwoven layers are denoted according to their constituent fibers (i.e., the letter) and fiber content (i.e., the digit). For example, N3/F5/L2 means the nonwoven layer is composed of 30 wt% of Nomex fibers, 50 wt% of flame-retardant PET fibers, and 20 wt% of LMPET fibers.

### 2.3. Preparation of Rigid Polyurethane (PU) Foam

Two-component rigid polyurethane foam is applied. Agent A (foaming agent) is made of Polyol, while agent B (hardener) consists of diphenylmethane diisocyanate (MDI). The two agents (1:1) are blended at 600 rpm for 10–30 s, and the resulting rigid PU foam has a density of 60 kg/m^3^.

### 2.4. Preparation of Flame-Retardant Polyurethane Foam

**Figure 1:**
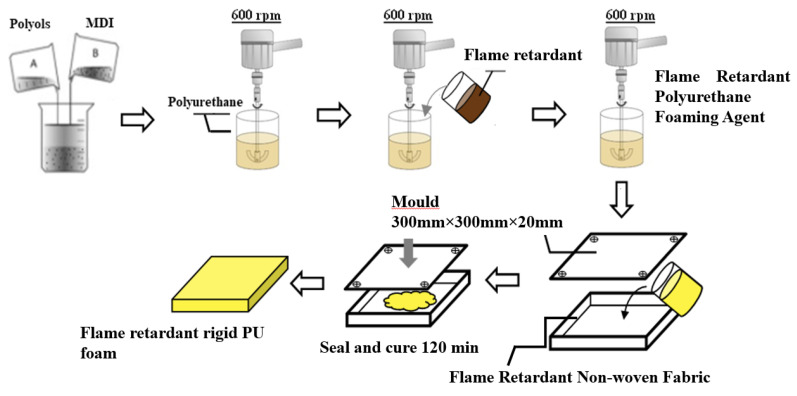
Diagram of the production process of rigid polyurethane foam.

Flame-retardant PU foam is constructed as described in Section 2.3 but with a different last stage. A flame-retardant nonwoven layer is mounted in a mold of 300 mm × 300 mm × 20 mm, after which 1 wt% of deionized water is first added to the mold, followed by the foaming blends. Next, the mold is sealed and stored at room temperature for 120 min. The use of deionized water aids in the foaming, the complete flow chart is shown in Figure 1. The resulting flame-retardant PU foam has a density of 60 kg/m^3^.

### 2.5. Preparation of Flame-Retardant Polyurethane Foam/Basalt Woven Fabric/Aluminized Foil Film Sandwich-Structured Composite Sheets

**Figure 2:**
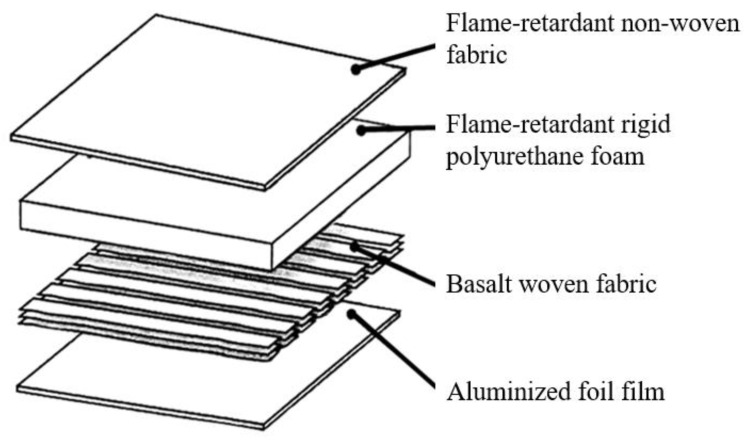
Diagram of sound-absorbent/heat-insulated/shock-proof/flame-retardant sandwich-structured composite sheets.

The sound-absorbent/shock-proof/flame-retardant sandwich-structured composite sheets are composed of the layers as specified in Figure 2, including a flame-retardant nonwoven layer, a flame-retardant rigid PU foam, a Basalt woven fabric, and an aluminized foil film.

### 2.6. Characterizations

Surface topography analysis is conducted using a cold field emission scanning electron microscope (HITACHI S3000N, Kyoto, Japan). The stretching test and tear test are conducted as specified in ASTM D5035 Maximum tensile breaking strength test standard using a universal strength testing machine (HT-2402, Hong Ta Co., ltd., Taichung, Taiwan). The bursting strength test is performed as specified in CNS 12,915 test standard. The compression performance test is conducted as specified in ASTM D1621-10 test standard using a universal strength testing machine (Instron 5566, US, Hong Ta Co., Ltd., Taichung, Taiwan). The piercing strength test is performed as specified in ASTM F1342 test standard. The impact resistance of samples is measured with drop weight impact testing machine (KN-001, Hong Ta Co., Ltd., Taichung, Taiwan) as specified in ASTM D4168-95 test standard. The limiting oxygen index (LOI) is measured with a limiting oxygen index analyzer (SMRI-010180, Dynisco Co., Ltd., Shanghai, China) as specified in ASTM D2863-13 test standard. The sound absorption coefficient is measured with a dual microphone impedance tube sound absorption tester (Hong Ta Co., Ltd., Taichung, Taiwan) as specified in ASTM E1050 (Dual Microphone Transfer Function Method). The electromagnetic interference shielding effectiveness (EMI SE) is measured as specified in ASTM D4935 using a shielding effectiveness test sample holder (EM-2107A, Hong Ta Co., Ltd., Taichung, Taiwan) and an electromagnetic wave generator (Advantest R-3132, Hong Ta Co., Ltd., Taichung, Taiwan). The number of tests per sample is 5.

## 3. Results and Discussion

### 3.1. Effects of Hot-Pressing Temperature and Needle Rolling Density over Mechanical Properties of Flame-Retardant NFL Nonwoven Fabrics

**Figure 3:**
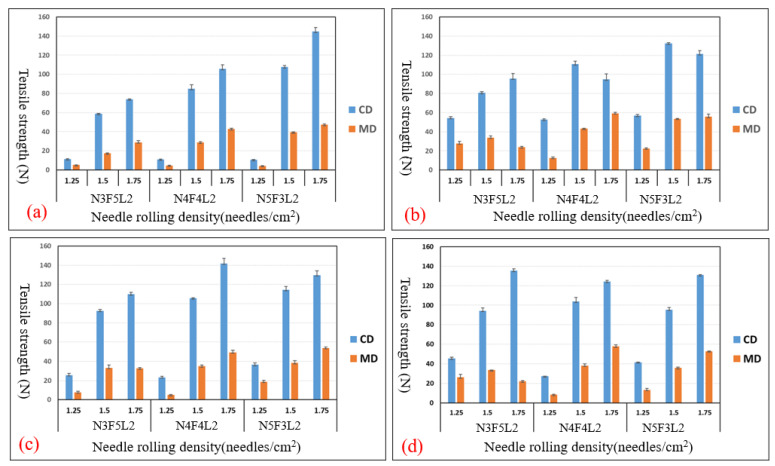
Tensile strength of (**a**) the control group and experimental groups—NFL nonwoven fabrics hot-pressed at (**b**) 140°C, (**c**) 150 °C, and (**d**) 160 °C.

**Figure 4:**
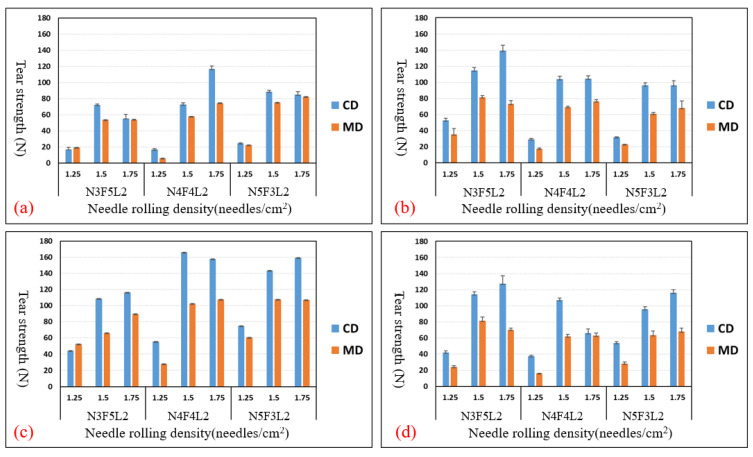
Tear strength of (**a**) the control group and experimental groups—NFL nonwoven fabrics hot-pressed at (**b**) 140 °C, (**c**) 150 °C, and (**d**) 160 °C.

**Figure 5:**
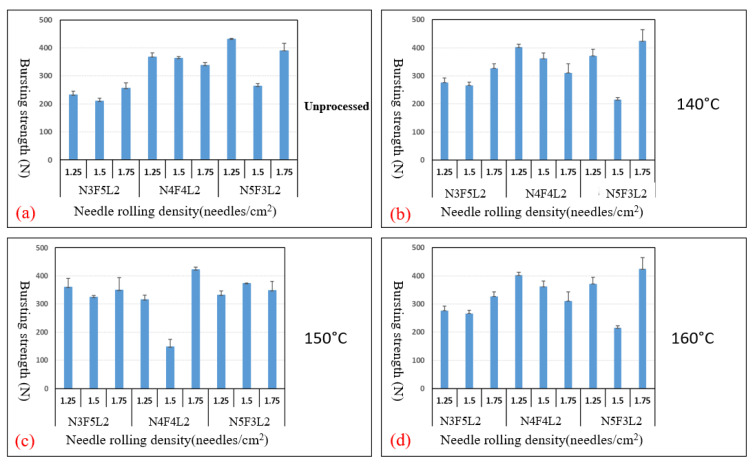
Bursting strength of (**a**) the control group and experimental groups—NFL nonwoven fabrics hot-pressed at (**b**) 140 °C, (**c**) 150 °C, and (**d**) 160 °C.

**Figure 6:**
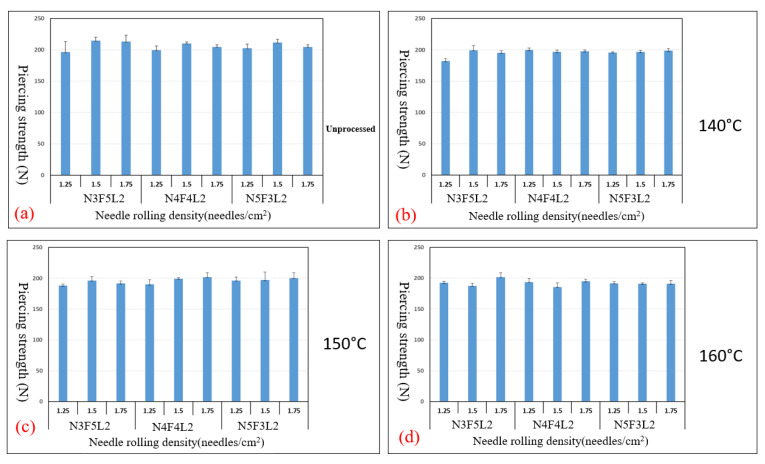
Piercing strength of (**a**) the control group, and experimental groups—NFL nonwoven fabrics hot-pressed at (**b**) 140 °C, (**c**) 150 °C, and (**d**) 160 °C.

Figure 3a–d show the tensile strength of different NFL nonwoven fabrics that receive needle rolling density of 1.25, 1.5, and 1.75 needles/cm^2^ and are hot-pressed at temperatures of 140 °C, 150 °C, and 160 °C. The control group is NFL nonwoven fabrics that are not hot-pressed. The optimal experimental group is N4F4L2 group, with needle rolling density of 1.75 needles/cm^2^ and hot-pressing temperature of 150 °C, as seen in Figure 3c. The optimal control group is N5F3L2 group, with the parameter of 1.75 needles/cm^2^, as seen in Figure 3a. Furthermore, Figure 4a–d show the tear strength of NFL nonwoven fabrics, where the optimal experimental group is the N4F4L2 group, with parameters of 1.50 needles/cm^2^ and 1.75 needles/cm^2^ as well as hot-pressing temperature of 150 °C, as shown in Figure 4c. Moreover, Figure 5a–d show the bursting strength of NFL nonwoven fabrics. The three optimal groups include N5F3L2 (140 °C, 1.75 needles/cm^2^), N4F4L2 (150 °C, 1.50 needles/cm^2^), and N5F3L2 (160 °C, 1.75 needles/cm^2^). Finally, Figure 6a–d show the piercing strength of NFL nonwoven fabrics. All groups exhibit comparable piercing strength, which proves that the piercing strength is not dependent on the hot-pressing temperature or needle rolling density. To sum up, according to the comprehensive mechanical evaluations, N4F4L2 (150 °C, 1.50 needles/cm^2^) is the optimal NFL nonwoven fabric.

### 3.2. Effects of Hot-Pressing Temperature and Needle Rolling Density over Combustion Performance of Flame-Retardant NFL Nonwoven Fabrics

**Figure 7:**
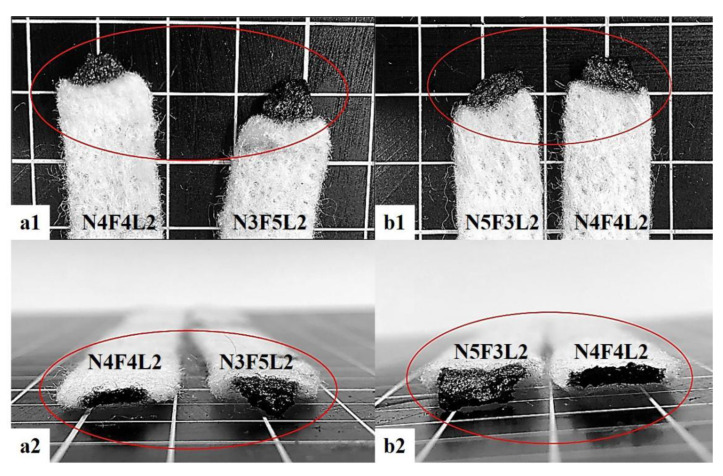
Combustion resistance of NFL nonwoven fabrics: (**a1**) burned area and (**a2**) corresponding cutting section of N4F4L2 and N3F5L2; (**b1**) burned area and (**b2**) corresponding cutting section of N4F4L2 and N5F3L2.

Table 1 shows the LOI of flame-retardant NFL nonwoven fabrics with regard to the blending ratio, needle rolling density, and hot-pressing (140 °C, 150 °C, and 160°C). The non-hot-pressed flame-retardant NFL nonwoven fabrics constitute the control group. In comparison, the experimental group and the control group demonstrate similar LOIs, suggesting that the combustion performance of samples is not correlated with hot-pressing temperature. Figure 7(a1,a2,b1,b2) compare the NFL nonwoven fabrics, where N3F5L2 and N5F3L exhibit a greater combustion area than that of N4F4L2. According to the combustion performance evaluation, the smaller the combustion area, the better the combustion resistance. Hence, N4F4L2 (150 °C, 1.50 needles/cm^2^) outperforms the other groups in terms of combustion resistance as well as mechanical properties, and this specified group is used for the subsequent discussions.

### 3.3. Property Evaluations of Flame-Retardant Polyurethane (PU) Foam

**Figure 8:**
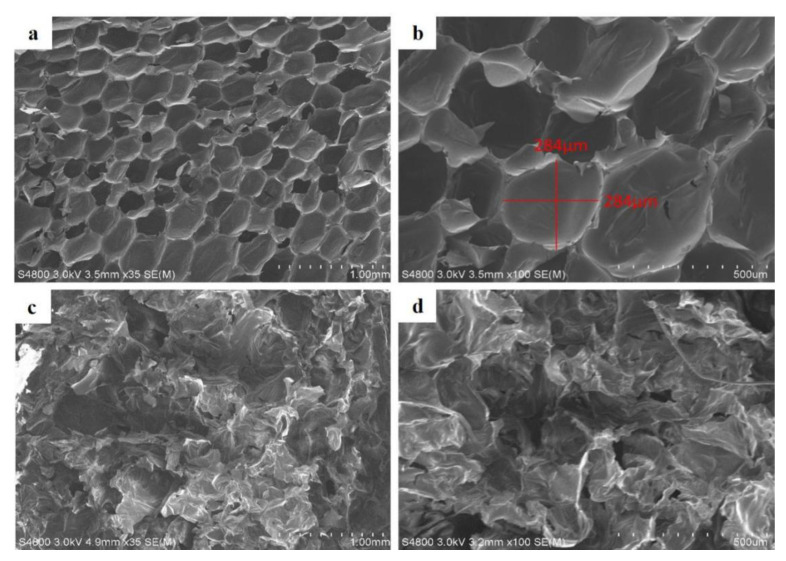
SEM images of flame-retardant rigid PU foam made without deionized water at (**a**) 35× and (**b**) 100× as well as with 1 wt% of deionized water at (**c**) 35× and (**d**) 100×.

Figure 8 shows the SEM images with a magnification of 35× and 100× of flame-retardant rigid PU foam. The cell morphology appears oval-shaped for the group without 1 wt% of deionized water as Figure 8a, which is also the typical cell morphology of rigid PU foam. The average cell size is 284 ± 3 µm, as seen in the image at 35× in Figure 8b. By contrast, Figure 8c,d show the SEM of flame-retardant rigid PU foam that contains 1 wt% of deionized water. Due to the presence of deionized water, the structural pattern is damaged, and the cells are almost not completely formed. Moreover, the presence of deionized water also leaves the curing PU foam in a sticky state because the flame retardant, hardener, and foaming agent are not foaming successfully. As shown in the curing rigid PU foam, the PU foaming blend is diluted because of deionized water, which leads to an agglomeration of the flame retardant and irregularly shaped cell chambers [30].

**Figure 9:**
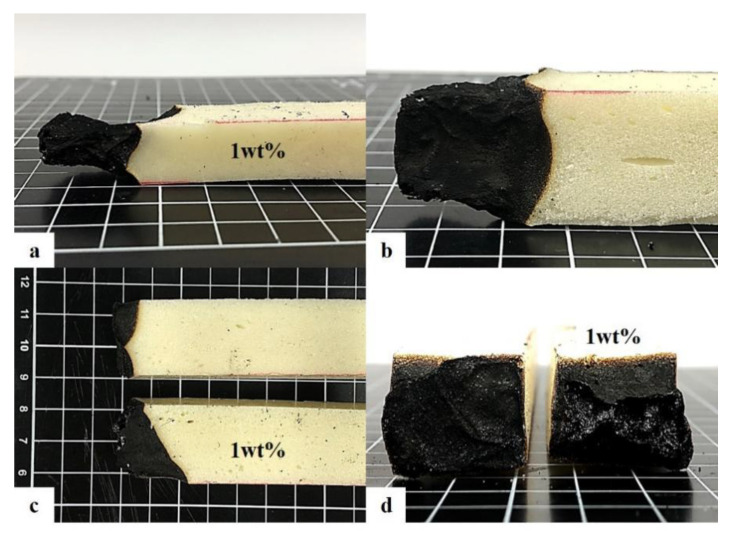
The lateral view of combustion area of flame-retardant PU foam that are incorporated (**a**) with and (**b**) without 1 wt% of deionized water. (**c**) The top view and (**d**) cutting section of combustion area of flame-retardant PU foam.

Although the incorporation of 1 wt% of deionized water results in uneven cell sizes and incomplete cell chambers, the difference in LOI of rigid PU foam is insignificant. The LOI for the group without the flame retardant is 15 ± 2; for the group with 10 wt% of flame retardant, it is 24 ± 3; and for the group that contains both 10 wt% of flame retardant and 1wt% of deionized water, it is 23 ± 2. After the combustion test, the side views indicate the damaged area of experimental group (with 1 wt% of deionized water) in Figure 9a is greater than the damaged area of the control group (without deionized water) in Figure 9b. When flame-retardant rigid PU foam is incorporated with 10 wt% of flame retardant and 1 wt% of deionized water, as illustrated in the top view in Figure 9c and the cutting section in Figure 9d, the LOI is not comparatively greater, but the damage area is increased by 10–15%.

### 3.4. Mechanical Properties of Flame-Retardant Nonwoven Fabric/Flame-Retardant Rigid PU Foam/Basalt Woven Fabric/Aluminized Foil Film Composite Sheets

**Figure 10:**
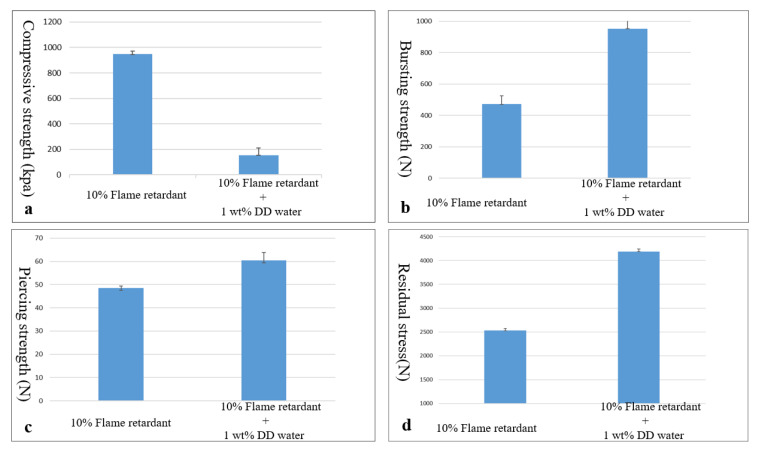
(**a**) Compressive strength, (**b**) bursting strength, (**c**) piercing strength, and (**d**) residual stress of flame-retardant nonwoven fabric/flame-retardant rigid PU foam/Basalt woven fabric/aluminized foil film composite sheets.

Figure 10a shows the compressive strength of flame-retardant nonwoven fabric/flame-retardant rigid PU foam/Basalt woven fabric/aluminized foil film composite sheets. The experimental groups contain 1 wt% of deionized water, while the control group does not contain deionized water. The control group has a considerably greater compressive strength than the experimental groups. The control group has no deionized water and, thus, represents dense and complete cell morphology, which is ascribed to the higher compressive strength. The experimental group contains 1 wt% of deionized water, which subsequently damages the cell morphology, and, therefore, the composite sheets show a comparatively lower compressive strength [31].

Figure 10b shows the bursting strength of flame-retardant nonwoven fabric/flame-retardant rigid PU foam/Basalt woven fabric/aluminized foil film composite sheets. The experimental groups demonstrate a greater bursting strength than the control group. The presence of 1 wt% of deionized water dilutes the mixture of flame retardant, hardener, and foaming agent, which inhibits the foaming level, and the foaming structural pattern is destroyed without forming complete chambers. When the experimental groups are exerted with a pressure, they tend to demonstrate ductility and bendability. As a result, the PU foam responds with a greater flexibility, which has a positive influence on the bursting strength.

Figure 10c shows the piercing strength of flame-retardant nonwoven fabric/flame-retardant rigid PU foam/Basalt woven fabric/aluminized foil film composite sheets. The experimental groups demonstrate a greater piercing strength than the control group. The incorporation of 1 wt% of deionized water hinders the foaming level and causes incomplete cell chambers in the PU foam. Furthermore, when PU foam is curing, it exhibits a sticky state that generates friction against the needle penetration in the piercing process. The agglomeration phenomenon in the PU foam also provides the PU foam with a greater thickness and depth that produce friction against the needle-like impactor; therefore, the experimental groups show greater piercing strength.

Figure 10d shows the residual stress of flame-retardant nonwoven fabric/flame-retardant rigid PU foam/Basalt woven fabric/aluminized foil film composite sheets. The constituent PU foam has a density of 60% kg/m^3^; here, the composite sheets are impacted by a 8.5 kg hammer that is released from a height of 250 mm to have a vertical free fall. The impact energy is approximately 20.83 J. The residual force for the control group and experimental groups are approximately 2500 N and 4200 N, respectively. For the experimental groups, the composite sheets are composed of irregularly shaped cell chambers in a convex–concave state due to the incorporation of 1 wt% of deionized water. When the hammer with a rounded tip strikes the composite sheets, the incomplete structure fails to absorb the impact energy evenly, and, therefore, the resulting residual force is higher than that of the control group.

### 3.5. Sound Absorption and EMI SE of Flame-Retardant Nonwoven Fabric/Flame-Retardant Rigid PU Foam/Basalt Woven Fabric/Aluminized Foil Film Composite Sheets

**Figure 11:**
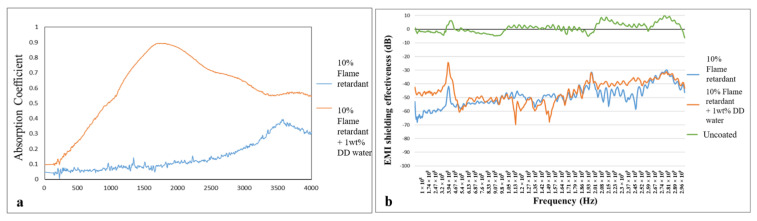
(**a**) Sound absorption capacity and (**b**) EMI SE of flame-retardant nonwoven fabric/flame-retardant rigid PU foam/Basalt woven fabric/aluminized foil film composite sheets.

Figure 11a shows that the control group (without deionized water) reflects with effective sound absorption value at a sound absorption frequency of 3400 Hz, and the sound absorption is decreased afterwards. The experimental group (with 1 wt% of deionized water) demonstrates effective sound absorption value at a sound absorption frequency of 600 Hz, and the sound absorption coefficient then achieves 0.9 when the frequency is 1500–2000 Hz. Afterward, the sound absorption coefficient declines to the minimal value of 0.5. To sum up, the control group demonstrates effective sound absorption at high frequencies, while the experimental groups demonstrate effective sound absorption at low frequencies. The presence of deionized water causes irregularly formed cell chambers in the PU foam, which can effectively block the acoustic energy and exhibit friction against a greater acoustic energy via refraction and reflection. As a result, the experimental groups are effective in absorbing sound waves at intermediate and high frequencies.

Figure 11b shows the EMI SE of composite sheets in relation to the presence of an aluminized foil film and 1 wt% of deionized water. Regardless of whether deionized water is incorporated, composite sheets without the aluminized foil film demonstrate EMI SE that is close to zero. Moreover, with or without the deionized water, composite sheets that contain an aluminized foil film exhibit EMI SE that exceeds 40 dB. It is proved that the efficient manufacturing process proposed in this study can highly improve the EMI SE of versatile composite planks.

### 3.6. Combustion Resistance of Flame-Retardant Nonwoven Fabric/Flame-Retardant Rigid PU Foam/Basalt Woven Fabric/Aluminized Foil Film Composite Sheets

**Figure 12:**
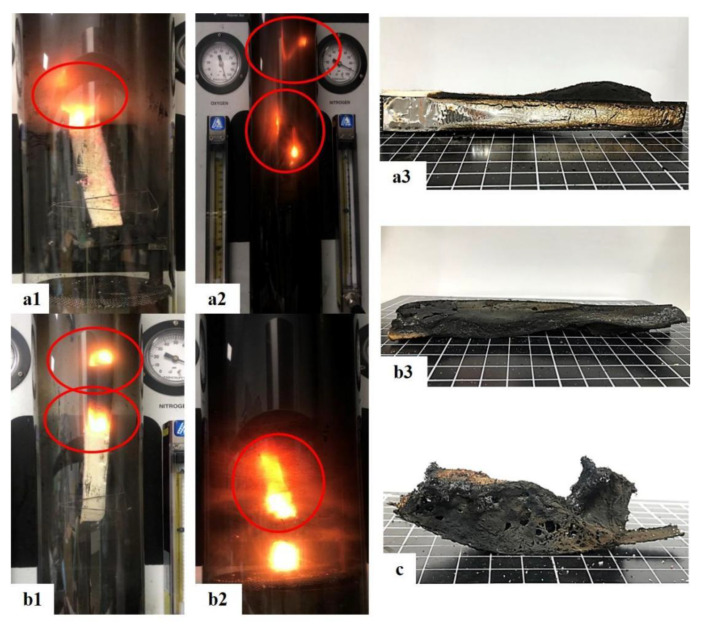
Combustion test for flame-retardant nonwoven fabric/flame-retardant rigid PU foam/Basalt woven fabric/aluminized foil film composite sheets (**a1**,**a2**) without and (**b1**,**b2**) with 1 wt% of deionized water. The samples that are set alight for (1) 5 s and (2) 30 s, and (3) samples that are burned until the flame dies. (**c**) Image of burned samples that do not consist of deionized water and Basalt woven fabric.

The composite sheets have 10 wt% of flame retardant in the constituent PU foam. The control group does not have deionized water, and the experimental groups contain 1 wt% of deionized water. The mechanical properties of the control group and the experimental groups differ and are associated with the cell morphology of flame-retardant rigid PU foam. Figure 12(a1) shows the control group, which has a lower level of clear fire source and less burn damage than the experimental group in Figure 12(b1). When the combustion lasts for 30 s, the flame gradually dies for the control group in Figure 12(a2), whereas the flames are still intense for the experimental groups, as shown in Figure 12(b2). After the fire is extinguished, the experimental groups are more severely damaged, carbonized, and distorted than the control group, as illustrated in Figure 12(a3,b3). However, when the Basalt woven fabric is removed from the composite sheets before the combustion test, the composite sheets that still contain flame-retardant nonwoven fabric and aluminized foil film are burnt, as shown in Figure 12c. In particular, the control group (without deionized water) shows severe carbonization and distortion after the combustion test. To sum up, the incorporation of Basalt woven fabric can mechanically strengthen the composite sheets while preventing the composite sheets from being rendered with distortion and deformation during the combustion test.

## 4. Conclusions

In this study, flame-retardant nonwoven fabric/flame-retardant rigid PU foam/Basalt woven fabric/aluminized foil film composite sheets are compared with regard to the incorporation of 1 wt% of deionized water in different tests. Without 1 wt% deionized water, the control group exhibits the maximum compressive strength. By contrast, the experimental group (with 1 wt% of deionized water) shows a greater piercing strength and impact strength along with excellent sound absorption capacity (0.9) at medium and low frequencies. The control group and the experimental groups attain an LOI of 26 and exhibit EMI SE that is greater than 40 dB due to the incorporation of aluminized foil film. The versatile composite sheets can be produced with an efficient manufacturing process, and they also acquire electromagnetic, wave shielding, and sound-absorbing functions without the polishing process for the surface. The reinforcement in EMI SE is attributed to the aluminized foil film, and the reinforcement in combustion resistance can be ascribed to the presence of Basalt woven fabrics, which can also reduce the damage area of the flame and reduce the generation of poisonous gas. In addition, the reinforcement in sound absorption is ascribed to the irregularly formed cell chamber of PU foam, which is a result of the incorporation of deionized water. This study develops and produces lightweight and versatile composite sheets that are recommended to be used with flexible purposes, such as building walls, floors, industrial factories, roofs, and interior decoration of vehicles. Adding aluminized foil film to the PU foam mold through simple processing can allow the electromagnetic wave shielding effect to be suitable for public places. If it is to achieve high frequencies, other electromagnetic wave shielding treatments are required. Secondly, although both nonwoven fabrics and PU foam have been processed with flame-retardant treatment, the burning gas is still poisonous; thus, this is an area of research that needs to be improved in the future.

## Figures and Tables

**Table 1:** LOI results of NFL nonwoven fabrics as related to blending ratio and needle rolling density.

**Unprocessed**				**140 °C**		
	**N3F5L2**	**N4F4L2**	**N5F3L2**	**N3F5L2**	N4F4L2	N5F3L2
**1.25**	23	22	25	23	26	26
**1.5**	22	24	24	23	24	26
**1.75**	23	26	25	24	24	25
**150 °C**				160 °C		
	N3F5L2	N4F4L2	N5F3L2	N3F5L2	N4F4L2	N5F3L2
**1.25**	24	24	23	22	24	24
**1.5**	23	23	23	24	23	24
**1.75**	23	23	23	22	23	24

## Data Availability

Not applicable.
